# Effect of Geometrical Asymmetry on the Phase Behavior of Rod-Coil Diblock Copolymers

**DOI:** 10.3390/polym8050184

**Published:** 2016-05-11

**Authors:** Jingying Yu, Faqiang Liu, Ping Tang, Feng Qiu, Hongdong Zhang, Yuliang Yang

**Affiliations:** State Key Laboratory of Molecular Engineering of Polymers, Collaborative Innovation Center of Polymers and Polymer Composite Materials, Department of Macromolecular Science, Fudan University, Shanghai 200433, China; 11210440027@fudan.edu.cn (J.Y.); 13110440007@fudan.edu.cn (F.L.); fengqiu@fudan.edu.cn (F.Q.); zhanghd@fudan.edu.cn (H.Z.); yuliangyang@fudan.edu.cn (Y.Y.)

**Keywords:** rod-coil block copolymers, geometrical asymmetry, SCFT, phase behavior

## Abstract

The effect of geometrical asymmetry β (described by the length-diameter ratio of rods) on the rod-coil diblock copolymer phase behavior is studied by implementation of self-consistent field theory (SCFT) in three-dimensional (3D) position space while considering the rod orientation on the spherical surface. The phase diagrams at different geometrical asymmetry show that the aspect ratio of rods β influences not only the order-disorder transition (ODT) but also the order-order transition (OOT). By exploring the phase diagram with interactions between rods and coils plotted against β, the β effect on the phase diagram is similar to the copolymer composition *f*. This suggests that non-lamellae structures can be obtained by tuning β, besides *f*. When the rods are slim compared with the isotropic shape of the coil segment (β is relatively large), the phase behavior is quite different from that of coil-coil diblock copolymers. In this case, only hexagonal cylinders with the coil at the convex side of the interface and lamella phases are stable even in the absence of orientational interaction between rods. The phase diagram is no longer symmetrical about the symmetric copolymer composition and cylinder phases occupy the large area of the phase diagram. The ODT is much lower than that of the coil-coil diblock copolymer system and the triple point at which disordered, cylinder and lamella phases coexist in equilibrium is located at rod composition *f*_R_ = 0.66. In contrast, when the rods are short and stumpy (β is smaller), the stretching entropy cost of coils can be alleviated and the phase behavior is similar to coil-coil diblocks. Therefore, the hexagonal cylinder phase formed by coils is also found beside the former two structures. Moreover, the ODT may even become a little higher than that of the coil-coil diblock copolymers due to the large interfacial area per chain provided by the stumpy rods, thus compensating the stretching entropy loss of the coils.

## 1. Introduction

In recent years, rod-coil diblock copolymers have increasingly attracted significant attention both in theory and in experiments as they can simultaneously show liquid crystalline behavior alongside microphase separation as coil-coil diblock copolymers [1,2]. The difference in chain topology and liquid crystal behavior fundamentally alters the physics of rod-coil systems and leads to a lot of phase structures not observed in coil-coil systems, such as wavy lamellar, zig-zag [3], arrowhead [3], perforated lamellar [3], hexagonal strip [4], puck structures [4,5] and so on. At the same time, transitions from the microphase separated state to liquid crystalline or isotropic states have been observed in both experiments and theories [6,7,8,9]. A wide variety of self-assembled nanostructures combined with liquid crystal behavior have provided a promising route for applications in organic electronics, biological molecules and high strength engineering resins.

To understand the physics of microphase separation, two parameters are introduced in a flexible AB diblock copolymer system including the isotropic Flory–Huggins interaction parameter χ*N* and the copolymer composition *f*, respectively [10,11]. In the rod-coil diblock copolymer system, two additional parameters are required to characterize the phase behavior due to the introduction of rod blocks. One is the anisotropic orientational interaction for the aligning of rigid blocks and the other is the geometrical asymmetry between rod and coil blocks for the difference in the scaling behavior. For flexible chains, only one parameter is required such as the Kuhn length for describing the chain size. However, rods need an additional parameter to describe the shape, such as the length to diameter ratio. The orientational interaction is generally described as the excluded volume interaction between the rod-like units that can be defined as the Maier–Saupe interaction μ*N* in most theoretical treatments [9,12,13,14]. It is due to the orientational interaction that rod-coil diblock copolymers can present liquid crystal behavior and form smectic A, smectic C structures and so on [7,8,9]. Furthermore, Song *et al.* found that rod-coil diblock copolymers can phase-separate, resulting from a strong enough orientational interaction even in the absence of χ*N* between rods and coils [12]. In fact, this orientation-induced phase separation phenomenon was also found in the experiments of ordered structures of the rod-coil system in the weak segregation limit (quite small value of χ*N*) [6,15]. 

In the rigid rod-coil diblock copolymer system, rod blocks can only align with each other in one direction but can neither be stretched like coils nor bended like wormlike chains because of the different chain topology on conformational entropy and molecular packing geometries. Moreover, rods and coils have different scaling behaviors as a function of molecular weight, resulting in a mismatch in size that is not captured by the coil volume fraction and requires the introduction of an additional parameter, namely geometrical asymmetry to characterize the ratio of the rod and coil block sizes. This parameter is defined in many cases as a ratio of the rod length *l* to the coil’s radius of gyration *R*_g_, which describes the geometrical shape of rods [6,9,10,11,12,13,14,15,16]. Therefore, it has a similar effect to the copolymer composition on the domain interface between the rod and coil block and thus the curved equilibrium microphase structures. The difference lies in the fact that the geometrical asymmetry is an independent parameter which includes the rod topology information in the three-dimensional (3D) space. 

The geometrical asymmetry can be generally tuned by changing one block or the total molecular weight and fixing the other at the same time in experiments, in which manner it can be applied to explain the different phase behavior of the rod-coil system with different molecular weights [3,6,7,16,17,18]. For example, Li *et al.* investigated the phase behavior of PMPCS-*b*-PS rod-coil diblock copolymers and found that tuning the asymmetry in the interfacial area through either decreasing the coil volume fraction or increasing the total molecular weight induced the phase transition from the perforated lamella to the lamella phase [3,17]. Olsen and Segalman found that in the moderately segregated PPV-*b*-PI rod-coil system, increasing the geometrical asymmetry as well as the composition asymmetry led to the formation of hexagonally packed phases of rectangular nanodomains in the coil-rich limit [7]. 

Previous theoretical studies paid more attention to the rod topology (or rod stiffness) effect on rod-coil diblock copolymer systems by simply assuming rod blocks with the same Kuhn length to be coils, which revealed the reduction in entropy due to the stiffness of rod blocks [19,20,21]. For example, Holyst and Schick firstly theoretically calculated the phase diagram in the weak segregation limit which was no longer symmetric around *f* = 0.5, and as a consequence the minimum of the stability curve was shifted to *f* = 0.45 [19]. After ignoring the orientational interaction to manifest the rod stiffness effect, Müller and Schick further pointed out that only structures with coils at the convex side of the rod-coil interface were stable by the self-consistent field equations through a partially numerical evaluation of the single chain partition function [20]. However, these results are not quantified in terms of changing the geometry asymmetry and did not take the rod diameter (geometrical shape of the rod) into consideration, which may have a profound effect on the phase behavior of rod-coil block copolymers.

Matsen and Barett calculated the phase behavior of rod-coil diblock copolymers in one-dimensional (1D) space by SCFT, in which the geometrical asymmetry parameter ν was firstly introduced, incorporating the effect of the rod diameter, which makes it possible to discuss the interface effect of rods and coils [8]. This parameter was defined as ν = *aN*^1/2^/*Nb*, where *a*, *b* and *N* are the coil Kuhn length, the rod Kuhn length and the total molecular weight, respectively. The definition is a little different from that in the experiments which create qualitative different results by changing the total molecular weight or coil volume fraction [7]. As *ν* increased from 0, it was found that the smectic A phase occupied a portion of the smectic C phase at the beginning and finally switched into the bilayer smectic A phase with a sufficiently high interfacial energy. Ganesan further extended this system in two-dimensional (2D) space and showed phase diagrams with ν = 0.15 and ν = 0.25, in which structures such as puck phases, arrowhead phases, and zig-zag phases were obtained and matched qualitatively with existing experiments [9]. However, the geometrical asymmetry effect on the phase behavior of the rod-coil diblock copolymer system was not particularly discussed as *ν* is still small enough to make the rod with slim conformation. 

It is now possible to study the rod-coil diblock copolymer system by using SCFT in 3D space thanks to the numerical method improvement on the solution of SCFT equations [22,23,24,25]. Kriksin *et al.* have recently reported a variety of structures in 3D space also by SCFT and particularly found one interesting morphology of hexagonally arranged chiral cylinders, which cannot be obtained in low-dimensional space [23]. Rods can choose a more suitable conformation in the 3D space of a computer simulation with less packing frustration which may help the rod-coil diblock copolymers to form into new structures. In addition, most previous works took rods as slim but rarely considered the case of podgy rods, where namely the rod diameter or the molecular weight effect on the phase behavior was ignored. As mentioned above, changing the geometrical asymmetry can change the interface of the rod and coil chains and thus influence the equilibrium structures, order–disorder transition (ODT) and order–order transition (OOT) of the system. Particularly, the geometrical effect can be hardly manifested if the orientational interaction is very large, as discussed in the previous work [9]. The orientational interaction is so large that the system tends to form into a large area of lamellar structures by rods aligning with each other. Therefore, we are going to include the rod geometry information and investigate the phase behavior of rod-coil block copolymers with 3D space numerical implementation of SCFT in this paper. To the best of our knowledge, this is the first time this issue is being considered. The full understanding of the geometrical asymmetry effect on the phase behavior will provide guidance to designing the self-assembled microstructures in experiments.

## 2. Theoretical Formalism

In this section, we briefly describe the SCFT theoretical framework for predicting the 3D thermodynamic equilibrium microstructures of rod-coil diblock copolymers. We consider incompressible rod-coil diblock copolymer melts consisting of *n* identical copolymer chains in volume *V*. Each coil block has *N*_C_ segments with their statistical segment length *a*, and each rod block has *N*_R_ segments with the length of each rod segment *b* = *l*_R_/*N*_R_, where *l*_R_ is the length of the rod block. For simplicity, the rod and coil segments are assumed as the same bulk number density $\rho_{0}$ = *nN*/*V*, where *N* = *N*_C_ + *N*_R_ denotes the copolymer chain length in each copolymer chain. Therefore, the volume fraction of the coil block is calculated as *f* = *N*_C_/*N* and *f*_R_ = 1 − *f* for the rod block. In the SCFT, the copolymer is parameterized with a continuous path variable *s* (in units of *N*) which increases from *s* = 0 at the beginning of the coil block, to *s* = *f* at the junction of the two blocks, and to *s* = 1 at the end of the rod block. The conformation of the coil block is described by the continuous Gaussian chain model, and the orientation of the rod block in the *k*th chain is described by a unit vector $u_{k}$ (starting from *s* = *f* to *s* = 1). 

The derivation, in detail, is similar to the SCFT for rod-coil diblock copolymer in bulk firstly described by Pryamitsyn and Ganesan [9]. The fundamental quantity to be calculated in mean field studies is the polymer segment probability distribution function q(r,s), representing the probability for a partial chain of length *s* ≤ *f* starting from the coil end (where *s* = 0) anywhere in the system and ending at position r. It satisfies a modified diffusion equation according to a flexible Gaussian chain mode1 but with the modified initial condition [9,26,27]. Here we only list the SCFT equations or the rod-coil diblock copolymers by the mean field approximation:

(1) $$\omega_{C}(r)=\chi N\phi_{R}(r)+\eta (r)$$

(2) $$\omega_{R}(r)=\chi N\phi_{C}(r)+\eta (r)$$

(3) $$\phi_{C}(r)+\phi_{R}(r)=1$$

(4) $$\phi_{C}(r)=\frac{1}{Q}\int_{0}^{f}dsq(r,s)q^{*}(r,s)$$

(5) $$\phi_{R}(r)=\frac{1}{Q\int du}\int_{0}^{1-f}ds\int duq(r-\beta su,f)\exp[\int_{0}^{1-f}ds^{\prime }\omega_{R}(r-\beta (s-s^{\prime })u)]$$

(6) M(r)=μNT(r)

(7) $$T(r)=\frac{1}{Q\int du}\int_{0}^{1-f}ds\int duq(r-\beta su,f)\exp[\int_{0}^{1-f}ds^{\prime }\omega_{R}(r-\beta (s-s^{\prime })u)](uu-\frac{I}{3})$$

(8) $$Q=\frac{1}{V}\int drq^{*}(r,f)$$

Equations (1)–(8) form a closed set of self-consistent equations, which can be solved numerically; $\phi_{C}(r)$ and $\phi_{R}(r)$ represent the density of coils and rods at location r, respectively. Orientational interactions between rods are described by a Maier-Saupe interaction energy in the mean field framework, where μ denotes the strength of the orientation interactions favoring the parallel alignment of rod blocks [28]. Then, χ*N* is the Flory–Huggins interaction parameter between chemicals unlike rod and coil segments which favors their microphase separation. T(r) is the orientational order parameter at location r; $\omega_{C}(r)$, $\omega_{R}(r)$ and M(r) are the potential field conjugated to the density of coils, rods and the orientational order parameter, respectively; η(r) ensures the incompressibility of the system; *Q* is the single chain partition function in the external fields. The free energy function in the unit of *nk*_B_*T* of the rod-coil diblock copolymer is given:

(9) $$\begin{array}{lll} F & = & \frac{1}{V}\int dr[\chi N\phi_{C}(r)\phi_{R}(r)-\omega_{C}(r)\phi_{C}(r)-\omega_{R}(r)\phi_{R}(r)\\ & & +\eta (r)(\phi_{C}(r)+\phi_{R}(r)-1)-\frac{\mu N}{2}T(r):T(r)+M(r):T(r)]-\ln Q \end{array}$$

The total free energy includes three parts: the Flory-Huggins internal energy *F*_inter_, the orientational interaction *F*_orien_ and the entropy −*TS*, which can be defined as [11]:

(10) $$F_{\text{inter}}=\frac{1}{V}\int dr\chi N\phi_{C}(r)\phi_{R}(r)$$

(11) $$F_{\text{orien}}=\frac{1}{V}\int dr[-\frac{\mu N}{2}T(r):T(r)+M(r):T(r)]$$

and the last term for entropy:

(12) $$-TS=-\ln Q+\frac{1}{V}\int dr[-\omega_{C}(r)\phi_{C}(r)-\omega_{R}(r)\phi_{R}(r)+\eta (r)(\phi_{C}(r)+\phi_{R}(r)-1)]$$

where $\beta =Nb/\sqrt{Na^{2}/6}=\sqrt{6}v^{-1}=\sqrt{6N}\times b/a$ is defined as the geometrical asymmetry between rod and coil blocks. This parameter can be tuned by changing the total molecular weight or the Kuhn length ratio. With the assumption of $\rho_{0}^{-1}$ = *a*^3^ = *bd*^2^, where *d* is the diameter of the rod, which can be used to describe the interfacial area between the rod and coil block for one diblock copolymer chain, β also can be written as:

(13) $$\beta =\sqrt{6N}\frac{a^{2}}{d^{2}}$$

It can be seen from Equation (13) that a small value of β corresponds to a short, stumpy rod conformation whereas a large value of β represents a slim rod conformation. The value of β can be chosen from 0 to infinity in principle. The value β = 10 chosen in this work corresponds to a representative experiment situation to show the phase behavior of slim rods in many works [9,29]; β = 1 is one of the typical values to show how the large interface per chain between rod and coil blocks effects the phase behavior of rod-coil blocks. In fact, it is difficult in experiments to synthesize copolymers with rod stumps enough. However, some systems such as nanoparticles with coils grafted onto the surface can be seen as an example of quite small β. To focus on the β effect on the phase structure, we only consider the condition of the absence of orientational interaction between rods. In this case, the SCFT equations listed above will be simplified by removing the calculation of M(r).

To numerically solve SCFT equations, the grid size of the space is chosen as Δ*x* = Δ*y* = Δ*z* = 0.25*R*_g_ and the discretization of contour variable *s* is chosen to be Δ*s* = 0.01 for coil segments. The orientation of rods is considered in 3D space (spherical surface), and the angular quadrature is still performed over an arbitrary unit vector u on the spherical surface. With the help of our previous paper, the spherical surface is discretized into icosahedron triangular mesh with *M* = 1442 vertexes to ensure accurate discretization of the angle distribution of rods [13,14,29]. The modified diffusion equation for a coil’s segment distribution q(r,s) is solved with the pseudospectral numerical method [30]. By using fast Fourier transforms to calculate integrals such as $\int_{0}^{1-f}ds\omega (r\pm \beta su)$ in Equations (5) and (7), the computation efficiency can be greatly improved [25]. The semi-implicit iteration scheme for solving the self-consistent field equations is also employed here to improve numerical efficiency and stability [22]. In order to obtain the relatively stable morphologies of the rod-coil diblock copolymer, we start the calculation with specified initial fields (several non-lamellae and lamellar structures in our manuscript) and compare the calculated converged energies in different initial field as well as with different lattice sizes. The phase transition points (ODT and OOT) at a fixed volume fraction in the diagram are obtained as the cross point of free energy curves at different initial fields by varying χ*N* with the increment of Δχ*N* = 0.1. The final stable phase structure is thus determined as the one with the lowest free energy. Furthermore, the pseudo-dynamical evolutions are carried out to a convergence of 10^−5^ in free energy and a convergence of 10^−4^ in field values. 

In most previous papers the orientational interaction was chosen as μ*N* = 4χ*N* to ensure the liquid crystalline phase behavior of the diblock copolymer system with rods aligning in a strong packing formation with each other during phase separation to form a flat interface, which is a lamellar structure [9,14]. Therefore, non-lamellar structures can only be obtained in a small area of the phase diagram in the rod-coil diblock copolymer system. In order to investigate non-lamellar structures of the rod-coil diblock copolymer system on a large scale, one better way is to decrease μ*N* to a low value. On one hand, it can help us to focus on the geometrical effect on the order-disorder transition of rod-coil diblock copolymers, where the orientational interaction is not so strong. On the other hand, the liquid crystal behavior is still kept when χ*N* becomes large. Therefore, at the beginning of this paper, we consider the system in a comparable orientational interaction with the interaction from immiscible blocks μ*N =* χ*N*, in which case the synergetic influence of *χN* and β on the phase behavior, especially in non-lamellar structures, can be conveniently investigated. Subsequently, we concentrate the geometrical asymmetry effect by ignoring orientational interaction. Furthermore, β = 10 and β = 1 are chosen, corresponding to slim and short, stumpy rod conformation, respectively, to study the geometry effect on the phase behavior of rod-coil diblock copolymer systems. 

## 3. Results and Discussion

### 3.1. Influence of Interactions (*χ*N and *μ*N) and *β* on the Phase Behavior

**Figure 1:**
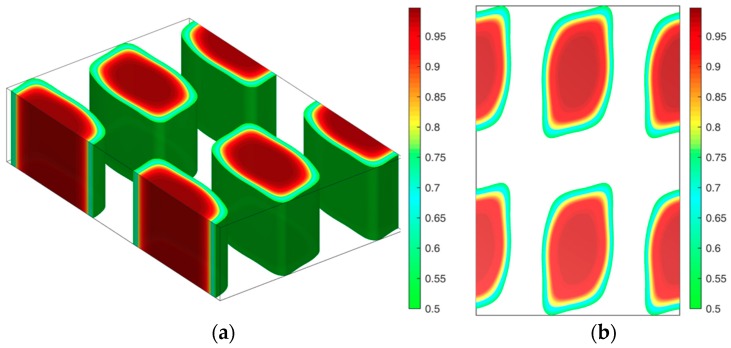
Rod puck phase with tetragonal symmetry of rod-coil diblocks at the condition of χ*N =* μ*N* = 14, β = 16 and *f* = 0.5. (**a**) 3D rod morphology view; (**b**) The corresponding density profiles for rods are plotted in a top view.

Previous studies reveal that β has little effect on the phase diagram of rod-coil diblock copolymer systems when the orientational interaction (μ*N*) dominates, such as μ*N = 4*χ*N* [9,12]. In this case, we found lamellae including smectic A and smectic C occupy most of the area of the phase diagram [31], and hexagonal cylinders with elliptical cross-sections formed by rods only occur at relatively high coil fractions and strong orientational interactions between rods [29]. When the orientational interaction between rods decreases to be comparable with χ*N*, however, the effect of β on the phase behavior can be manifested as the orientational interaction being weak near the ODT and the liquid crystal behavior hardly observed. For example, Tang *et al.* reported a series of non-lamellar structures including body-centered cubic, A15, hexagonal and Gyroid phases by ignoring μ*N* in the rod-coil diblock copolymer systems [32]. They also calculated these OOT phase boundaries at several different β values [32]. Therefore, non-lamellae structures can be obtained depending on the β and μ*N*. In order to distinguish the effect of the relation between μ*N* and β on the phase behavior, we first calculate the phase diagram of χ*N versus* β at *f* = 0.5 and *f* = 0.65 under the condition of μ*N =* χ*N*. For each certain fixed composition, the phase diagram is calculated by plotting the free energy curves of various competitive phases by varying χ*N* with the increment of Δχ*N* = 0.1. The corresponding χ*N* of the intersection point of these curves is set as the phase boundary point. Although different initial fields, including hexagonal cylinders, four structures such as complex bicontinuous structures (Gyroid), tetragonal cylinders, body-centered spheres and A15, found by Tang *et al.* in the absence of interactions between rods [32] and lamellae are included during calculations, only HEX (coil), HEX (rod) phases, and lamellae (LAM) in this work are obtained by altering β. The HEX (coil) structure is hexagonal cylinders formed by coil blocks, LAM represents lamellae and HEX (rod) is hexagonal cylinders formed by rod blocks. With further increasing β, puck cylinders with tetragonal symmetry formed by rods, shown in Figure 1, occur in a quite narrow area of the phase diagram, as shown in Table 1. This is similar to the tetragonal cylinders found in our previous study and the puck phase found by us and Pryamitsyn and Ganesan [9,13,14].

**Figure 2:**
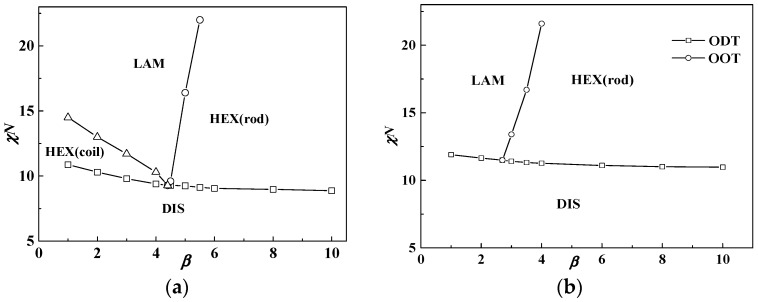
Phase diagrams of rod-coil diblock copolymers in β *versus* χ*N* (μ*N* = *χN*). (**a**) *f* = 0.5; (**b**) *f* = 0.65. The symbol box and circle (triangle) represent the point of order-disorder transition (ODT) and order-order transition (OOT), respectively.

Figure 2a shows the phase diagram at coil volume fraction *f* = 0.5, in which condition coil-coil diblock copolymers develop into the lamellar phase with χ*N*_ODT_ = 10.5 [10,11,33]. We note that the phase diagram is only within the range of β ≤ 10 for saving computational tasks, and puck cylinders with tetragonal symmetry in Figure 1 formed by rods occur only in a narrow area of the phase diagram with a further increasing β (see Table 1). The triple point where lamellar, cylinder and disordered phases coexist in equilibrium is located at about β = 4.4 with χ*N*_ODT_ = 9.3. When β > 2, the χ*N*_ODT_ < 10.5. This is due to the fact that rods do not have conformation entropy so the introduction of rod blocks will decrease the system energy and thus decrease χ*N*_ODT_, compared to the coil blocks. Therefore, the χ*N*_ODT_ of rod-coil blocks is between 10.5 for coil-coil blocks and 6.1 for rod-rod blocks [34]. Interestingly, we note that the χ*N*_ODT_ continuously increases as β decreases and even exceeds 10.5 of the coil-coil diblock copolymer system when β < 2. For example, the χ*N*_ODT_ at β = 1 and *f* = 0.5 is 10.9, larger than 10.5 of the coil-coil diblock copolymer system at the same volume fraction. In this case, the short, stumpy rod can provide a larger interfacial area per chain for coils to release stretching entropy, thus showing a slightly higher χ*N*_ODT_ than that of coil-coil blocks. This can be further illustrated by comparing the domain period near the ODT in different cases including coil-coil, rod-coil and rod-rod diblock copolymers. The domain period from rod-coil blocks is *D* = 0.75*bN* = 3.25*aN*^0.5^ when β = 4.4, *f* = 0.5 and χ*N*_ODT_ = 9.5, which is smaller than that of rod-rod diblock copolymers (*D* = 0.91*bN*) but much larger than that of coil-coil diblock copolymers (*D* = 1.32*aN*^0.5^) [33,34]. 

In addition, when the coil volume fraction is increased to *f* = 0.65, the phase diagram changes a lot as illustrated in Figure 2b. In this condition, the HEX (coil) structure disappears and the triple point moves to about β = 3, which is smaller than that in Figure 2a. This demonstrates that rod-coil diblocks tend to form into structures with coils at the convex interface, namely HEX (rod) near the ODT. This is because the coil conformational entropy dominates the whole free energy when the volume fraction of coils is larger than that of rods. Therefore, the HEX (coil) structure will not come up even when β is very small and is replaced by the LAM structure. Our previous study for semiflexible-coil block copolymers also found hexagonal packed elliptical domains formed by rods at a coil volume fraction of 0.7 with a strong orientational interaction by numerical implementation of SCFT in 2D space [13]. In order to decrease the total free energy, one better way for the system is that coils stay at the convex side of the interface to decrease entropy loss. On the other hand, it also can be seen from Figure 2 that increasing χ*N* can lead the phase transition from cylinders to lamellae structures no matter what the value of β is. As in this part μ*N =* χ*N*, and increasing χ*N* will lead rods to align with each other, thus favoring liquid crystal behavior with lamellae structure.

In conclusion, the phase diagram in Figure 2 is quite similar to that in the plot of χ*N*(μ*N*) *vs. f*. This demonstrates that copolymer composition *f* and conformational asymmetry between two blocks β have a similar effect on the phase behavior. Therefore, non-lamellar structures for rod-coil blocks (anisotropic orientational interactions between rods favor liquid crystal behavior, thus resulting in the majority of the phase diagram being occupied by the layered structures) can also be obtained by changing β, besides *f*. Except to obtain non-lamellar structures (HEX (rod)) in a large coil volume fraction condition as shown in Figure 2b, Figure 2a shows that changing β can also result in non-lamellar structures including HEX (coil) and especially HEX (rod) in a case of comparable volume fractions between rods and coils, and even quite large rod fractions.

**Figure 3:**
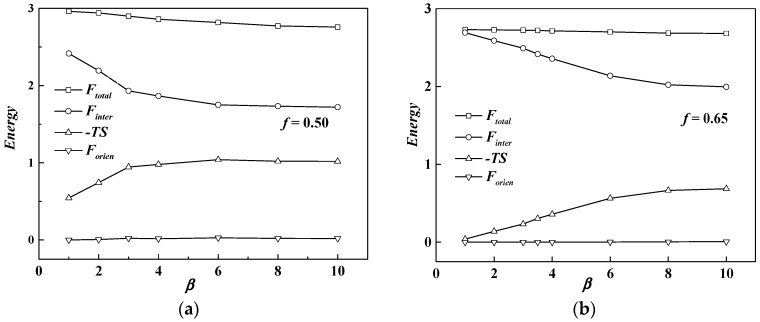
Free energy *F*_total_ and three of its contributions, *F*_inter_, *F*_orien_ and −*TS* in Equations (10)–(12) at χ*N* = 12 as a function of β. (**a**) *f* = 0.5; (**b**) *f* = 0.65.

To obtain a thorough understanding of the phase behavior of the rod-coil diblock copolymer system, the free energy *F*_total_ and three of its contributions mentioned in the theoretical part, the Flory–Huggins interaction *F*_inter_, the entropy loss −*TS* and the orientational interaction *F*_orien_ at χ*N* = 12 as a function of β, are drawn in Figure 3, in which Figure 3a,b represent *f* = 0.5 and *f* = 0.65, respectively. It is shown that the total free energy increases with the decrease of β, which means the free energy of the lamellar phase at a smaller β is higher than that of the cylinder phase formed at a larger β. The order-disorder transition point χ*N*_ODT_ is consequently increased, as discussed in Figure 2, with increase of the total free energy. It also can be seen that the Flory-Huggins interaction energy makes the main contribution to the whole free energy of the system, especially at a smaller value of β or a larger value of the coil volume fraction, while the entropy loss behaves in a converse way. As we set μ*N =* χ*N*, the orientational interaction energy is close to zero to make little contribution to the free energy of the system. This is the reason that many works choose μ*N* = 4χ*N* to ensure liquid crystal behavior for the rod-coil diblock copolymer system.

When β decreases from 10 to 1, the rod changes from a slim conformation to a short, stumpy one. In the condition of *f* = 0.65 and β = 10, rods are slim with a small diameter, thus providing narrow interfacial area for coils, resulting in coils much more stretched and far away from the rods, just like polymer brushes at the flat interface [35,36]. In order to decrease the system energy, coils prefer to stay at the convex side of the rod-coil interface to decrease the entropy loss, leading to the formation of rod-rich cylinders, HEX (rod). Compared with coil-coil diblocks with the same cylinder phase formed, the free energy of rod-coil diblock copolymers can be smaller due to the lack of conformation entropy of the rods. As a consequence, the χ*N*_ODT_ of rod-coil blocks is lower than that of coil-coil blocks. As mentioned above, the interaction energy dominates the free energy as β decreases. Therefore, it is the main driving force to bring the increase of the χ*N*_ODT_ at relatively small β. Particularly the Flory–Huggins interaction energy between rods and coils increases so much after phase transition from cylinder to lamella that the χ*N*_ODT_ increases a lot and even exceeds that of the coil-coil diblock copolymer system. For example, χ*N*_ODT_ = 10.9 at β = 1 and *f* = 0.5 is larger than the 10.5 value of the coil-coil diblock copolymer system at the same volume fraction.

### 3.2. Effect of *β* When the Orientational Interaction Is Turned off

According to the above discussion, the decrease of geometrical asymmetry β affects the phase transition by increasing the interfacial tension for compensating the stretching entropy loss of coils. To further focus on the β influence on the phase behavior, the orientational interaction is ignored in this part. It is noted that we only consider HEX (rod) and HEX (coil) as the representative non-lamellar structures and lamellae as candidate structures for simplicity to concentrate on the β effect on the non-lamellae of the rod-coil system. Two conditions including β = 10 and β = 1 are chosen to investigate the phase behavior for rods with slim and short, stumpy conformations, respectively. 

**Figure 4:**
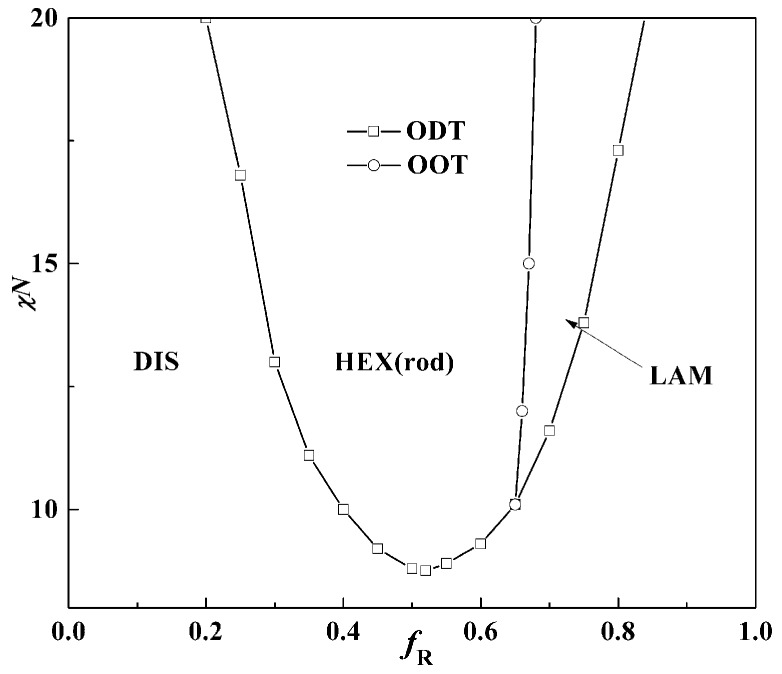
Phase diagram of 3D structures for rod-coil diblock copolymers with β = 10 without orientation interaction. LAM and HEX (rod) represent the lamella and hexagonal cylinder formed by rods, respectively. The symbol box and circle represent the point of order-disorder transition (ODT), and order-order transition (OOT), respectively.

In Figure 4, the phase diagram for β = 10 as a function of the rod volume fraction *f*_R_ and Flory-Huggins interaction χ*N* is firstly presented as a comparison with the result by Müller and Schick [20]. The phase diagram is slightly asymmetric as the critical point of χ*N*_ODT_ = 8.7 is at about *f* = 0.48 (*f*_R_ = 0.52), which is lower than the typical value χ*N*_ODT_ = 10.5 of coil-coil diblock copolymers. Two kinds of structures: lamella (LAM) and hexagonal cylinders aggregated by rod blocks (HEX (rod)) come up at the phase diagram and the triple point where LAM, HEX (rod) and DIS coexist is located at about *f* = 0.35 (*f*_R_ = 0.65). The hexagonal cylinders formed by rods occupy the majority of the phase diagram, leaving a small area for the lamellar phase. These results are almost the same as the work of Müller and Schick, who also pointed out that stable structures are only those with coils at the convex side of the rod-coil interface [20]. Figure 2 also shows that a larger value of β can lead to the formation of HEX (rod), even at quite large rod volume fractions. Kriksin *et al.* also found that only such non-lamellar structures are observed with rods forming the core and coils located at the outside convex surface [25]. It seems that increasing β or *f* can both induce the non-lamellar structure HEX (rod) and HEX (coil) formation, respectively. 

**Figure 5:**
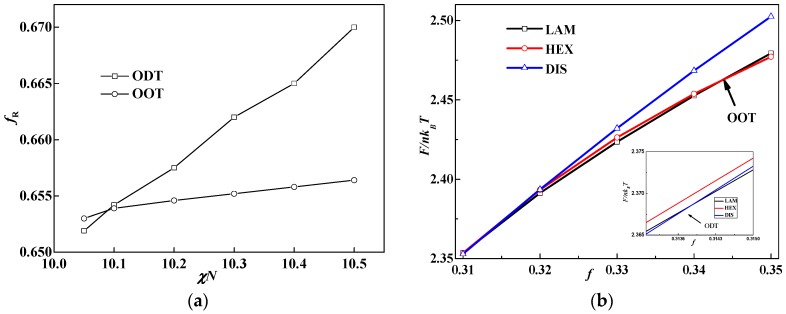
(**a**) Triple point (the cross point of OOT (HEX(rod) to LAM) and ODT) calculations of rod-coil blocks at β = 10 and χ*N* = 11; (**b**) Free energy as a function of coil volume fraction in different phases: lamella (LAM), cylinder (HEX) and disorder (DIS) phase. The inset in (**b**) is the locally amplified section near the cross point of ODT.

The difference lies in the result that the triple point is located at about *f* = 0.35 (*f*_R_ = 0.65) in our current work, while the triple point calculated by Müller and Schick coincides with the lowest point of ODT [20]. Our calculations show that the triple point location depends on the values of β for rod-coil block copolymers. The triple point of the phase diagram is calculated as the cross point of OOT (cylinder structure to LAM) and ODT (disorder structure to LAM) by comparing the free energy of the lamella and cylinder with the disorder phase as shown in Figure 5. Matsen even theoretically examined the effect of two coil statistical segment length ratio $a_{A}/a_{B}$ on the phase behavior of the coil-coil diblock copolymers and found the triple point moved to the component side with the larger Kuhn length [37]. When β = 10, the length ratio of the rod to coil is very large, which can be seen as the limit of the Kuhn length ratio $a_{A}/a_{B}$ in the coil-coil diblock copolymer system. The interface therefore tends to curve towards the rod blocks, allowing the coil blocks to relax at the convex side for decreasing the stretching entropy penalty. Kriksin *et al.* also found that the phase diagram is asymmetric and the lamellar structures exist even at quite asymmetric compositions, due to conformational asymmetry between two coil blocks [38,39]. Moreover, the majority component with a large Kuhn length can aggregate into micelle inner cores with a minority component-forming matrix, different from conventional coil-coil diblocks.

**Figure 6:**
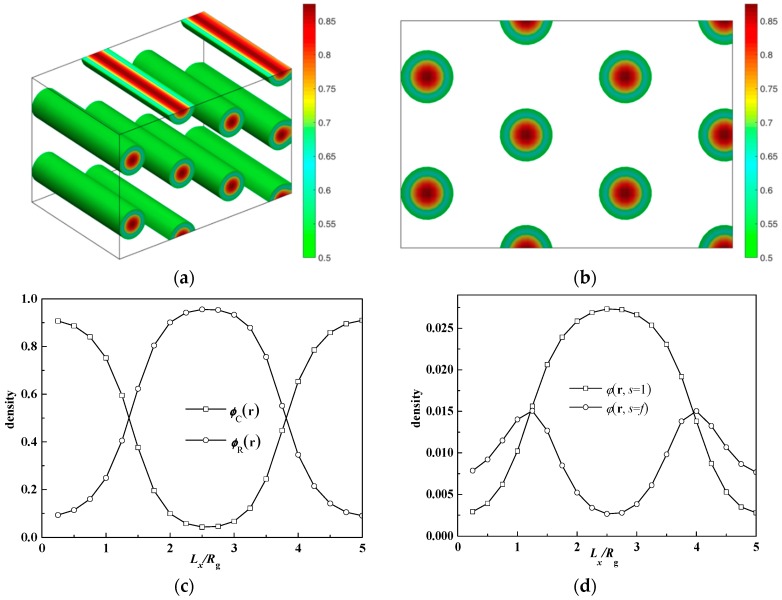
(**a**) Hexagonal cylinders formed by coils of rod-coil diblocks at χ*N* = 14, μ*N* = 0, β = 10 and *f* = 0.7. (**a**) 3D rod morphology view; (**b**) The corresponding density profiles for rods are plotted in a top view; (**c**) HEX (rod) phase density profile along one rod domain in (**b**); (**d**) Normalized density profile of terminal (ϕ(r,s=1)) and junction points (ϕ(r,s=f)) of the rod along one rod domain.

To illustrate the cylinder structure, the morphology and the density profile are given in Figure 6 at β = 10 and *f* = 0.7. Figure 6a,b show the 3D structure and the cross-section at the XZ plane, respectively. The lattice corresponding to the lowest energy here is 5*R*_g_ × 8.5*R*_g_ which means the structure is in hexagonal asymmetry. To show this structure in more detail, the density profile of the rod and coil component, across one rod domain as well as the rod terminal φ(r,s=1) and junction points ϕ(r,s=f), are also shown in Figure 6c,d. It can be seen that the rod blocks aggregate in the center of the rod-rich domain, leaving the link distributed at the rod-coil interface. The domain size in this parameter is 2.75*R*_g_, while the rod length can be calculated from the equation as *l*_R_ = *Nbf*_R_ = β*R*_g_*f*_R_ = 3*R*_g_. We can calculate the orientational tensor field S(r)=uu−I/3 and its maximum eigenvalue $\lambda_{\text{max}}\left(r\right)$, the latter of which shows the orientation degree of the rod block. Thus, $\lambda_{\text{max}}\left(r\right)$ is 0.002 at the point with the largest rod density, which means rods do not show long-range orientation due to the ignorance of the orientational interaction between rods. Therefore, the domain morphology cross-section develops into the same circle, such as in the case of the coil-coil block copolymer system. In the study of the 2D puck phase of coil-semiflexible diblock copolymers, Gao *et al.* pointed out that the orientation of semiflexible blocks resulted in the ellipse domain morphology [29]. Our results reconfirm that the puck phase is due to the anisotropic orientational interaction between semiflexible blocks. In the rod-coil diblock copolymer system, orientational interaction will generally decrease the whole system energy by aligning rod blocks so as to form a flat interface such as smectic A or smectic C, which are obtained in many works and in our previous work [12,13,14,29]. When the orientational interaction is ignored, the geometrical asymmetry effect on the phase behavior of the rod-coil diblock copolymer system is manifested. For β = 10, the rod is long and thin so the interface between the rod and coil blocks per chain is small. Coils are thus much more stretched away from the interface, leading to more entropy penalty. In this condition, rod-rich cylinders with coils at the convex side of the interface can provide more space for coils to relax. The consequence is that a large area in the phase diagram comes with rod-rich cylinders. 

**Figure 7:**
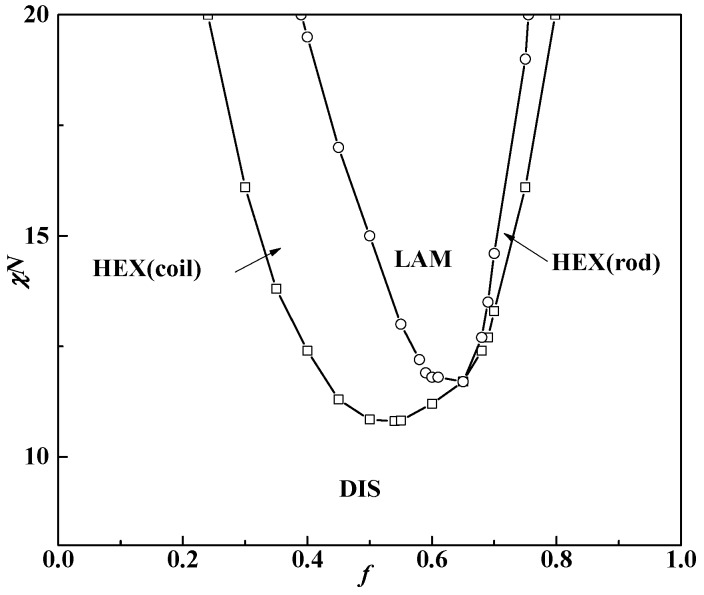
Rod-coil diblock copolymer 3D bulk phase diagram when β = 1 without orientation interaction. LAM and HEX (rod) and HEX (coil) represent the lamellar and hexagonal cylinders formed by rods and hexagonal cylinders formed by coil phases, respectively. The symbol box and circle represent the point of order-disorder transition (ODT) and order-order transition (OOT), respectively.

When β decreases to 1, however, the phase diagram changes a lot. As illustrated in Figure 7, the whole phase diagram is shifted with the lowest *χN*_ODT_ point at *f* = 0.54 and *χN*_ODT_ = 10.8, which is slightly higher than the value of coil-coil diblock copolymers. This is also in accordance with the result in the condition of μ*N* = χ*N*, as discussed in Figure 2 when β = 1. It is widely thought that rod-coil diblock copolymers have relatively strong phase-separation abilities so χ*N*_ODT_ should be lower than that of coil-coil diblock copolymers in the same volume fraction. Our result, however, shows a different phenomenon: that the rod-coil diblock copolymer with extremely small β is more difficult to phase-separate than coil-coil diblock copolymers. As mentioned above, a smaller β means the rods are in short, stumpy conformation. Compared with β = 10, in this case rods can provide enough interface for coils with much more flexible conformation. The entropy penalty with a small β is therefore less than that with a large β, but the Flory–Huggins interaction becomes very large. The order-disorder transition behavior of the rod-coil diblock copolymer system is thus much more similar to the coil-coil diblock copolymer system. 

Besides the above two structures LAM and HEX formed by rods, cylinders formed by coil blocks (HEX (coil)) come up when both *f* and χ*N* are small. The triple point moves to about *f* = 0.65 and χ*N*_ODT_ = 11.8. The lamellar and cylinder phases formed by coil blocks occupy the most area of the phase diagram and the cylinder phase formed by rod blocks only comes up in a relatively small area. Stupp and coworkers have observed rod-rich hexagonal cylinder and lamella phases in thin films of rod-coil diblock molecules by TEM and found the transition between the two ordered phases to occur at lower rod contents [40]. 

**Figure 8:**
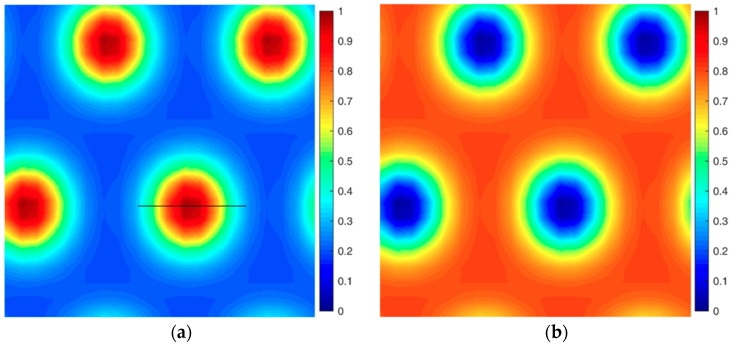

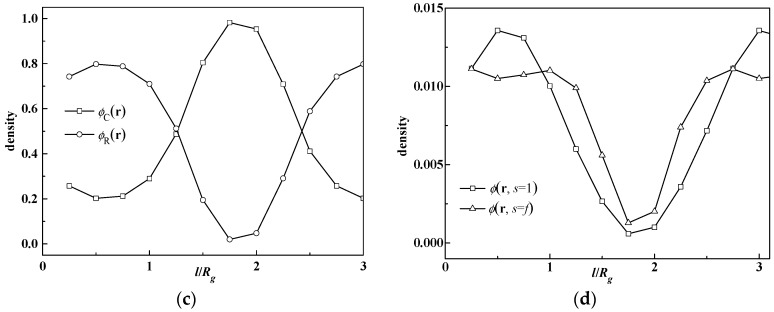
Density profile of HEX (coil) phase at *f* = 0.35 and *β* = 1. (**a**) Coil density distribution; (**b**) Rod block density distribution; (**c**) Density profiles for rods and coils along the coil domain donated by the black solid line in (**a**); (**d**) Rod head (*s* = 1) and link (*s* = *f* = 0.35) points density profile along the coil domain.

Figure 8 presents the density profile for the cylinder phase formed by coil blocks. The domain size of the cylinder is calculated at about 1*R*_g_, the same as the coil size in scale of the whole molecular weight *N*. Although coils aggregate into ordered cylinder phases, there is still a certain amount of coils in the rod domains. This means that in this condition, rods do not align very closely so that coils can be dispersed around rods. This kind of structure has been also reported recently by Shi *et al.* in PDMS-*b*-PMPCS rod-coil diblock copolymers [16]. The copolymer is annealed at 125 °C lower than the liquid crystalline formation temperature, and namely the orientational interaction is weak. In that condition, the rod block PMPCS is amorphous and the coils would rather form hexagonal cylinders. Matsen also reported that a significant concentration of coil segments accumulated at the center of the rod domains when he studied the liquid crystal phase behavior of rod-coil diblock copolymers in 1D space [8]. This is because the interface between the rod and coil block per chain will increase as β decreases, and the coils would prefer to be absorbed near the surface rather than extended far away for less conformation entropy loss. Alternative ways of reducing the high interfacial energy are to include a finite compressibility or another component in the system, such as a selective solvent for the rod blocks. 

**Figure 9:**
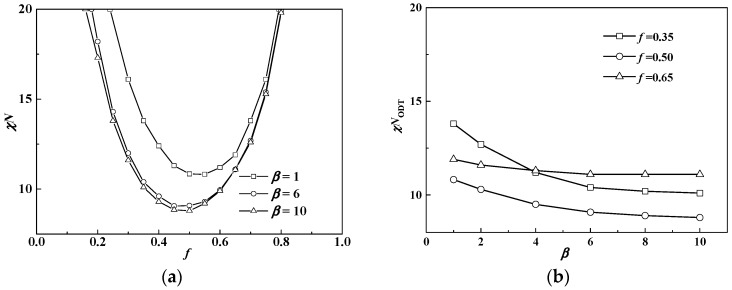
(**a**) Phase diagrams at β = 1, 6, and 10; (**b**) χ*N*_ODT_ at different β values.

If the rod has a large diameter *d*, providing larger interfacial area per chain and thus favoring the coil, it is easy to fulfill the conformation with little entropy loss. In contrast, if the rod is slim with a small diameter *d*, the coil block will be excluded much further from the interface, resulting in a larger coil conformation entropy loss. To illustrate the effect of β, the order-disorder transitions at β = 1,6,10 are shown in Figure 9a. It can be seen that the ODT increases as β decreases, especially when the rod volume fraction dominates. This phenomenon is also demonstrated in Figure 9b by comparing χ*N*_ODT_ at different values of *f* and β. For example, when the rod volume fraction is large, *f*_R_ = 0.65, and the χ*N*_ODT_ will increase dramatically at small β.

**Figure 10:**
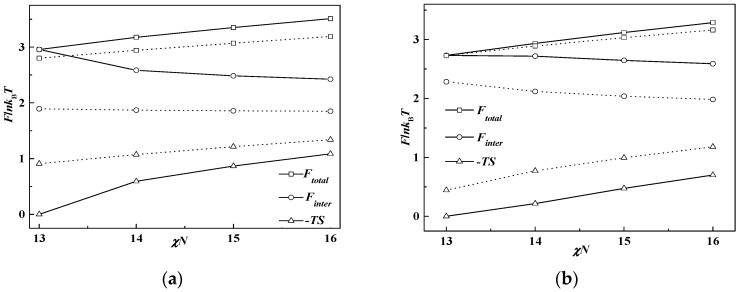
Free energy *F*_total_ interaction energy *F*_inter_ and entropy loss −*TS* as a function of interaction strength. (**a**) *f* = 0.35; (**b**) *f* = 0.7. The solid and dotted line represents β = 1 and β = 10, respectively.

As the orientational interaction is set as 0, the total energy *F*_total_ is composed of the Flory-Huggins interaction energy *F*_inter_ and the coil conformation entropy −*TS*. In Figure 10, *F*_total_, *F*_inter_ and −*TS* are plotted as a function of χ*N* at *f* = 0.35 and *f* = 0.7 for the two different β values, respectively. It can be seen that decreasing β will increase the whole system energy and the gap between two β values will be enlarged if the rod block volume fraction is larger than that of the coil block. We also observe that the interaction energy contributes much more to the whole free energy, demonstrating that in this condition, the system is dominated by the interfacial area between rod and coil blocks. This is because when β is small, short, stumpy rods offer large interfacial area for decreasing the coil’s stretching entropy loss. The Flory-Huggins interaction in this way becomes large, but with small entropic penalty. As χ*N* becomes large, coils need to stretch much more in a larger interfacial area per rod chain than that with a large β with a small interface. This accordingly results in the phase separation at a much higher value of χ*N*. 

## 4. Conclusions

In this article, the effect of geometrical asymmetry between rods and coils on the rod-coil diblock copolymer system is investigated. It is found that the geometrical asymmetry β plays an important role in the phase behavior of the rod-coil diblock copolymer system. When μ*N* = χ*N*, increasing β leads to the transition from HEX (coil) to LAM and LAM to HEX (rod), along with the decrease of χ*N*_ODT_. When μ*N* = 0, the phase diagram is naturally asymmetric for the rod-coil diblock copolymer system at the lowest point of order-disorder transition, which increases from *f* = 0.48 (*f*_R_ = 0.52), χ*N*_ODT_ = 8.7 at β = 10 to *f* = 0.54 (*f*_R_ = 0.46), χ*N*_ODT_ = 10.7 at β = 1. Furthermore, the whole phase diagram is not in bilateral symmetry corresponding to the lowest ODT point. The graph is lower at the left and higher at the right at β = 10 while this situation inverses to lower at the right and higher at the left at β = 1. Thirdly, the triple point corresponds to *f* = 0.35 at β = 10 and *f* = 0.65 at β = 1, neither of which coincides with the lowest point of the order-disorder transition point χ*N*_ODT_. 

The results show that β also has a great influence on the order-order transition (OOT) of rod-coil diblock copolymer systems. When rods are slim, such as β = 10, two kinds of structures, lamellae and hexagonal cylinders, come up at the phase diagram with cylinder phases occupying most of the area. The cylinder phases are formed by the rod blocks with coil blocks at the convex side of the surface when the coil volume fraction is larger than 0.35. In contrast, when β = 1, the phase behavior is much more similar to that of the coil-coil diblock copolymer system. 

The geometrical asymmetry effect on the phase behavior of rod-coil diblock copolymer systems stems from the competition of the coil entropy loss and the interaction between two kinds of blocks. Decreasing *β* will dramatically increase the Flory–Huggins interaction but decrease the entropy loss. Compared with coil-coil diblock copolymer systems, the slim rod lacks entropy conformation and coils tend to extend away from the rod-coil interface. The former results in the decrease of χ*N*_ODT_ and the latter leads to a large area of rod-rich cylinders in the phase diagram. On the other hand, the short, stumpy rod can offer a large interfacial area per chain and thus for the coil in a more flexible conformation. Although the interaction energy will be increased, flexible coils make the system much more similar in the phase behavior to coil-coil diblock copolymer systems. 

In the end, different definitions are employed in experiments for the geometrical asymmetry of the rod-coil diblock copolymer system. In this work, β includes the information of the rod Kuhn length, rod diameter and molecular weight. To make one comparable result in the experiments, modifying the molecular weight, for example, is a good choice for phase behavior studies. Then we can discuss the molecular weight effect, which may be used to explain the different phase behaviors of oligomers and high molecular weight rod-coil diblock copolymer systems. 

## Figures and Tables

**Table 1:** 3D structures summary of rod-coil diblock copolymers at μ*N* = χ*N* and *f* = 0.5.

β	10	12	14	16	18
*χN* = 10	HEX (rod)	HEX (rod)	HEX (rod)	HEX (rod)	HEX (rod)
*χN* = 12	HEX (rod)	HEX (rod)	HEX (rod)	HEX (rod)	HEX (rod)
*χN* = 14	HEX (rod)	HEX (rod)	Puck	Puck	Puck
*χN* = 16	LAM	LAM	LAM	LAM	LAM
*χN* = 18	LAM	LAM	LAM	LAM	LAM
